# Neonatal hypoxic ischemic encephalopathy increases acute kidney injury urinary biomarkers in a rat model

**DOI:** 10.14814/phy2.15533

**Published:** 2022-12-21

**Authors:** Angela M. Groves, Carl J. Johnston, Gisela G. Beutner, Jane E. Dahlstrom, Mark Koina, Michael A. O'Reilly, George Porter, Patrick D. Brophy, Alison L. Kent

**Affiliations:** ^1^ Department of Pediatrics University of Rochester School of Medicine and Dentistry New York Rochester USA; ^2^ Department of Radiation Oncology University of Rochester School of Medicine and Dentistry New York Rochester USA; ^3^ Division of Cardiology University of Rochester School of Medicine and Dentistry New York Rochester USA; ^4^ Department of Anatomical Pathology, ACT Pathology Canberra Health Services Canberra Australia; ^5^ College of Health and Medicine Australian National University Canberra Australia; ^6^ Division of Nephrology, University of Rochester School of Medicine and Dentistry Golisano Children's Hospital at University of Rochester Medical Center New York Rochester USA

**Keywords:** AKI, HIE, urinary biomarkers

## Abstract

Hypoxic ischemic encephalopathy (HIE) is associated with acute kidney injury (AKI) in neonates with birth asphyxia. This study aimed to utilize urinary biomarkers to characterize AKI in an established neonatal rat model of HIE. Day 7 Sprague–Dawley rat pups underwent HIE using the Rice–Vannucci model (unilateral carotid ligation followed by 120 mins of 8% oxygen). Controls included no surgery and sham surgery. Weights and urine for biomarkers (NGAL, osteopontin, KIM‐1, albumin) were collected the day prior, daily for 3 days post‐intervention, and at sacrifice day 14. Kidneys and brains were processed for histology. HIE pups displayed histological evidence of kidney injury including damage to the proximal tubules, consistent with resolving acute tubular necrosis, and had significantly elevated urinary levels of NGAL and albumin compared to sham or controls 1‐day post‐insult that elevated for 3 days. KIM‐1 significantly increased for 2 days post‐HIE. HIE did not significantly alter osteopontin levels. Seven days post‐start of experiment, controls were 81.2% above starting weight compared to 52.1% in HIE pups. NGAL and albumin levels inversely correlated with body weight following HIE injury. The AKI produced by the Rice–Vannucci HIE model is detectable by urinary biomarkers, which can be used for future studies of treatments to reduce kidney injury.

## Graphical abstract

Although animal models of brain injury resulting from HIE are well established, the kidney injury that occurs is not well studied. This study aims to characterize AKI in an established rat model of HIE and track injury progression using sensitive urinary biomarkers. This provides an experimental approach utilizable for testing effectiveness of therapies that treat AKI.

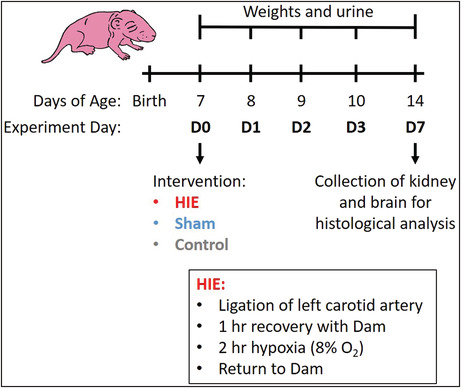

## 1 INTRODUCTION

Perinatal asphyxia resulting in hypoxic ischemic encephalopathy (HIE) occurs in 2‐5 per 1000 live births and is a major cause of morbidity and mortality (Pfister & Soll, 2010). HIE is one of the multiple etiologies that comprise the clinical syndrome of neonatal encephalopathy and is specific to hypoxia–ischemia. Perinatal asphyxia results in multi‐organ dysfunction involving the kidneys, as well as the brain. Neonatal acute kidney injury (AKI) occurs in up to 40% of neonates with HIE and is an independent risk factor for increased duration of ventilation, length of stay, poor neurodevelopmental outcome, and mortality (Karlowicz & Adelman, 1995; Kirkley et al., 2019; Sarkar et al., 2014; Selewski et al., 2013). There is growing evidence that an episode of AKI in the neonatal period results in increased risk of chronic kidney disease in later life (Chaturvedi et al., 2017; Harer et al., 2017). Although studies on the use of hypothermia treatment for HIE found reductions in the degree of AKI (Tanigasalam et al., 2016; van Wincoop et al., 2021), many of the observations were short term, and evidence of renal dysfunction remained present and was apparent in later childhood (Robertsson Grossmann et al., 2022), highlighting the continuing need for effective preventative strategies.

The Rice–Vannucci neonatal rat HIE model has been widely used to examine the impacts of different potential treatments to reduce the neurological damage associated with HIE in human neonates. This model initiates HIE at 7 days of age, at time traditionally considered to be equivalent to a term human infant, based originally off of measurements of tissue weight; later advancements, however, expanded considerations to include benchmarks in cell proliferation and maturation (Semple et al., 2013). This more integrated assessment resulted in a model system that places the human equivalence at post‐conception day 260, which is in the late pre‐term to term time frame (Workman et al., 2013). In rats, postnatal day 7 is also an age that corresponds to a sensitive window of kidney development that more mimics late pre‐term humans, as it is prior to the cessation of nephrogenesis. Nephrotoxicity at this early stage has been linked to kidney dysfunction later in life (Seely, 2017). While two studies have recently examined whether this model results in AKI, it remains less well studied. Wang et al. showed that acetyl‐l‐carnitine prevented the decrease in renal organic cation/carnitine transporter 2 and pyruvate dehydrogenase levels at 24 h after injury which would improve energy metabolism in the kidney (Wang et al., 2019). Xu et al. found that melatonin reduced expression of edema‐related proteins, including aquaporin‐4, zonula occludens‐1, and occludin following hypoxic ischemic insult (Xu et al., 2017). Given the incidence of AKI resulting from perinatal asphyxia, our study therefore aimed to further expand understanding of the renal pathology produced by this model. We hypothesized that urinary biomarkers can be utilized to identify and track the progression of HIE‐induced AKI.

Serum creatinine is currently the gold standard for diagnosing AKI; however, it is estimated that >50% of renal function is lost before a rise in creatinine is observed. The sensitivity of the test therefore underestimates the extent of injury. Urinary biomarkers of AKI are being developed that are more sensitive as at detecting kidney injury. This sensitivity is critical for diagnosing and determining treatment for early or mild to moderate renal injury and loss of function, and highlights the utility of urinary biomarkers in experimental models AKI. Urinary biomarkers NGAL (neutrophil gelatinase‐associated protein), kidney injury molecule 1 (KIM‐1), and osteopontin (OPN) were shown to trend higher in neonates with AKI, as defined by a rise in serum creatinine (Askenazi et al., 2016; Rumpel et al., 2022). The NGAL gene is found to be upregulated in very early kidney injury and is a highly induced protein in the kidney after ischemic or nephrotoxic AKI in animal models (Devarajan, 2015; Devarajan et al., 2003; Mishra et al., 2003; Mishra et al., 2004; Supavekin et al., 2003). The expression of albumin is greatly induced above typical levels in the kidney following AKI in both animal models and clinical studies (Ware et al., 2011). Furthermore, the application of biomarkers in this model enables it to be utilized to examine treatments that may reduce AKI, particularly when associated with perinatal HIE.

## 2 METHODS

### 2.1 Animals

This study was approved by the University of Rochester's Institutional Animal Care and Use Commitee (IACUC) (102314/2019‐30). National Institutes of Health guidelines were complied with in the care and handling of the animals. Timed‐pregnant Sprague Dawley rats were obtained from Charles River Laboratories and were housed and cared for in the central animal facility. All dams and pups received identical standard husbandry conditions as provided by our institution's vivarium staff and the dams received standard chow and water ad libitum. Dams delivered litters that contained roughly equal numbers of pups (10–12 pups per litter). To ensure even distribution of pups between each experimental condition, equal numbers of male and female pups were selected from each of three litters and were assigned an experimental group. This process was replicated for each group and each group contained roughly equal numbers of pups from each litter.

### 2.2 Surgery and hypoxia–ischemia

**FIGURE 1:**
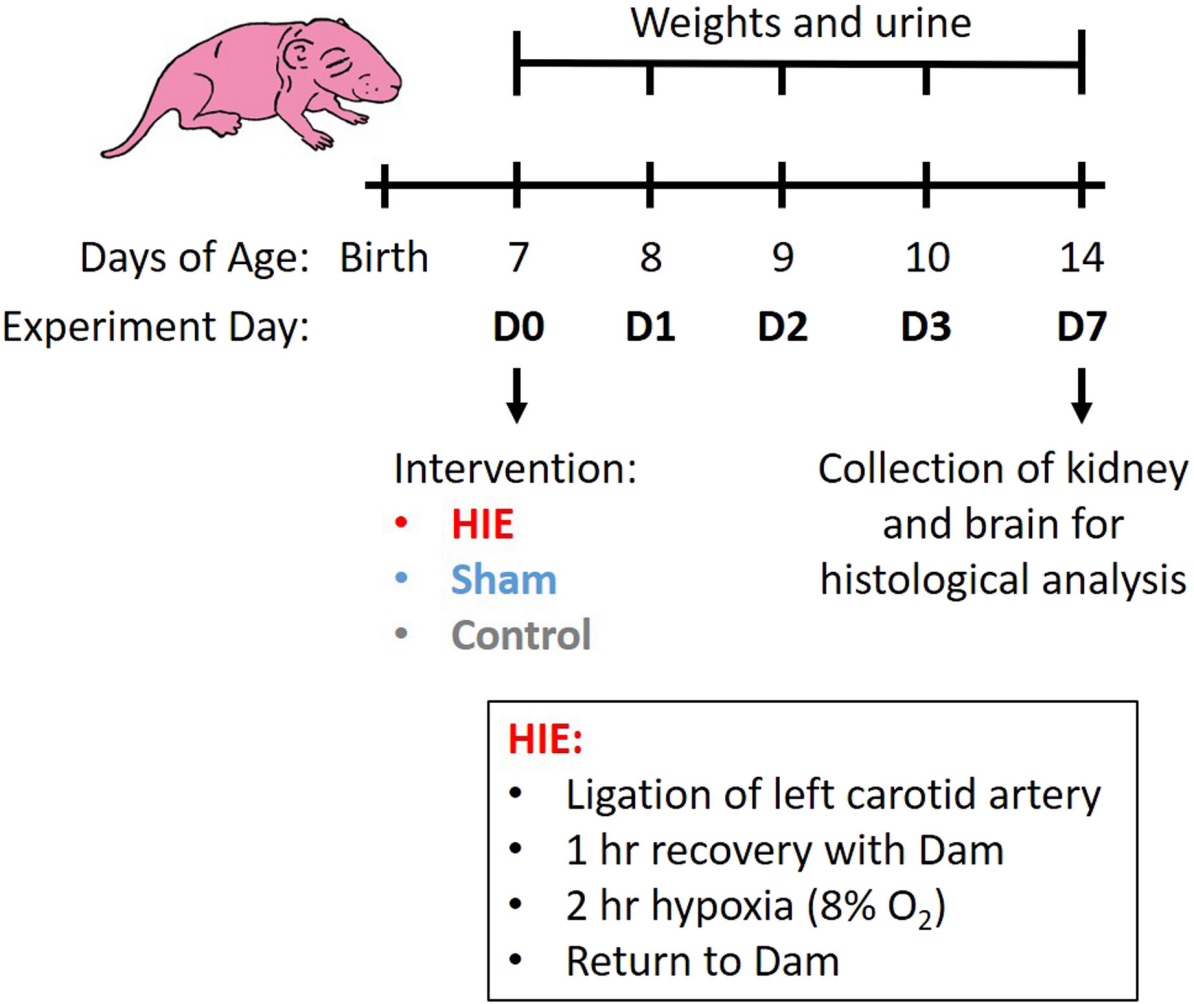
Rice–Vannucci model of HIE and sample collection schedule. HIE: Seven‐day‐old Sprague–Dawley rat pups underwent surgery for ligation of the left carotid artery, recovery for 1 h with their dam, then were placed in a hypoxia chamber at 8% oxygen for 120 min and returned to their dam to recover. Temperature was maintained throughout at 37 degrees. Controls: no surgery or anesthesia. Sham: Anesthesia and skin incision. Sample collection: Daily weights and urine were collected prior to intervention on D0, post‐intervention on D1‐3, and at sacrifice, D7.

Seven‐day old rat pups, equivalent to late preterm or term human neonates (Workman et al., 2013) were anesthetized with 2% isoflurane, analgesia provided was subdermal buprenorphine. The rat pups underwent a modification of the Rice–Vannucci model (Rice et al., 1981; Vannucci & Vannucci, 1997, 2005) (Figure 1) which involved ligation of the left carotid artery and recovery for 1 h before the pups were placed in a hypoxia chamber at 8% oxygen for 120 min. Temperature was maintained throughout at 37^C^ degrees by placing pups on isothermal pads designed to maintain a constant temperature for several hours. Pups were then returned to the dam to recover and feed as normal. Controls included a group that did not receive surgery or anesthesia as well as a group that received sham surgery with anesthesia. Sham surgery was comprised of skin incision and exposure of the left carotid artery. Pups were weighed daily for the first 3 days post‐intervention and on day of sacrifice.

### 2.3 Urine collection and biomarker analysis

Urine was collected by gently scruffing the pup, by grasping gently along the nape of the neck and down the back, and holding the pup upright to induce urination. Freely expelled urine was collected directly into a 1.5‐ml microfuge tube. Care was taken to prevent the tube from contacting the pup itself or any fecal matter also expelled during this process to minimize contamination. Volumes greater than 50 μl were considered adequate for analysis. Urine was collected the day before intervention, daily for 3 days post‐intervention and on the day of sacrifice. Urine was centrifuged at 300*g* to remove particulates, filtered with a 0.2‐micron sterile filter and frozen at −80^C^. A multiplex ELISA kit, Meso Scale Discovery Rat Kidney Injury Panel V Plex Assay (catalog no. K15162C; Meso Scale Discovery) measuring NGAL, osteopontin, KIM‐1 and albumin was used to detect these analytes in urine, which was diluted 1:10 and the assay performed according to manufactures instructions. Each plate required 20 μl of urine per sample. Standard duplicates showed good reproducibility having a low average signal confidence of variability (CV), (average sample CV% = 2.9647).

### 2.4 Sample processing and histology

At postnatal day, 14 pups were sacrificed with Euthasol (100 mg/kg; Virbac) followed by exposure of abdominal and chest organs by midline incision. Vasculature was cleared by injection into the right atrium with heparin sodium (1 unit/gram body weight), papaverine hydrochloride (1.2 mg dose), and 0.9% sodium chloride (until perfusate ran clear). Tissues were then perfusion fixed with 2.5% glutaraldehyde in 0.1 M phosphate buffer.

#### 2.4.1 Kidney histology

The right kidney was excised and immersed in 2.5% glutaraldehyde in phosphate buffer for 5 h then transferred to 10% buffered formalin for 48 h then placed in 70% EtOH until processed for paraffin embedding which included bisection along midline axis through the hilum and placing both cut surfaces placed face down into the cassette. Kidney sections were stained with Hematoxylin and Eosin (H & E) and examined by an experienced histopathologist (JED). Full cross sections of each half of the kidney were examined that included the cortex, medulla, and renal pelvis of each pup.

#### 2.4.2 Brain histology

Whole brains were excised and placed in 10% formalin for 10 days for complete fixation then placed in 70% EtOH until processed. Prior to paraffin embedding permanent black histology dye was used to identify the left hemisphere post‐processing. The cerebrum was separated and cut into three sections: fronto, parietal, and parieto‐occipital areas, and the same distance between the cuts was maintained in each animal. The brain stem and cerebellum were separated from each other and bisected longitudinally. Brain sections were stained with H&E and examined by a histopathologist (JED). Whole mount sections were examined to compare both cerebral hemispheres and the hippocampus. The striatum and thalamus were not assessed.

### 2.5 Statistics

GraphpadPrism 9.4 software was used for the statistical analyses (Graphpad Software) of urinary markers. Data are presented as mean ± SEM. Statistical significance was determined by two‐way ANOVA followed by Sidak's multiple Comparison Test when ANOVA showed significant differences. Reported comparisons include between HIE and control or sham groups at each time point. For correlations between biomarker values and body weight, data were fit using least squares regression and R squared values were calculated to quantify goodness‐of‐fit. For comparison significance a sum of squares F test was used. A value of *p* < 0.05 was considered significant.

## 3 RESULTS

**FIGURE 2:**
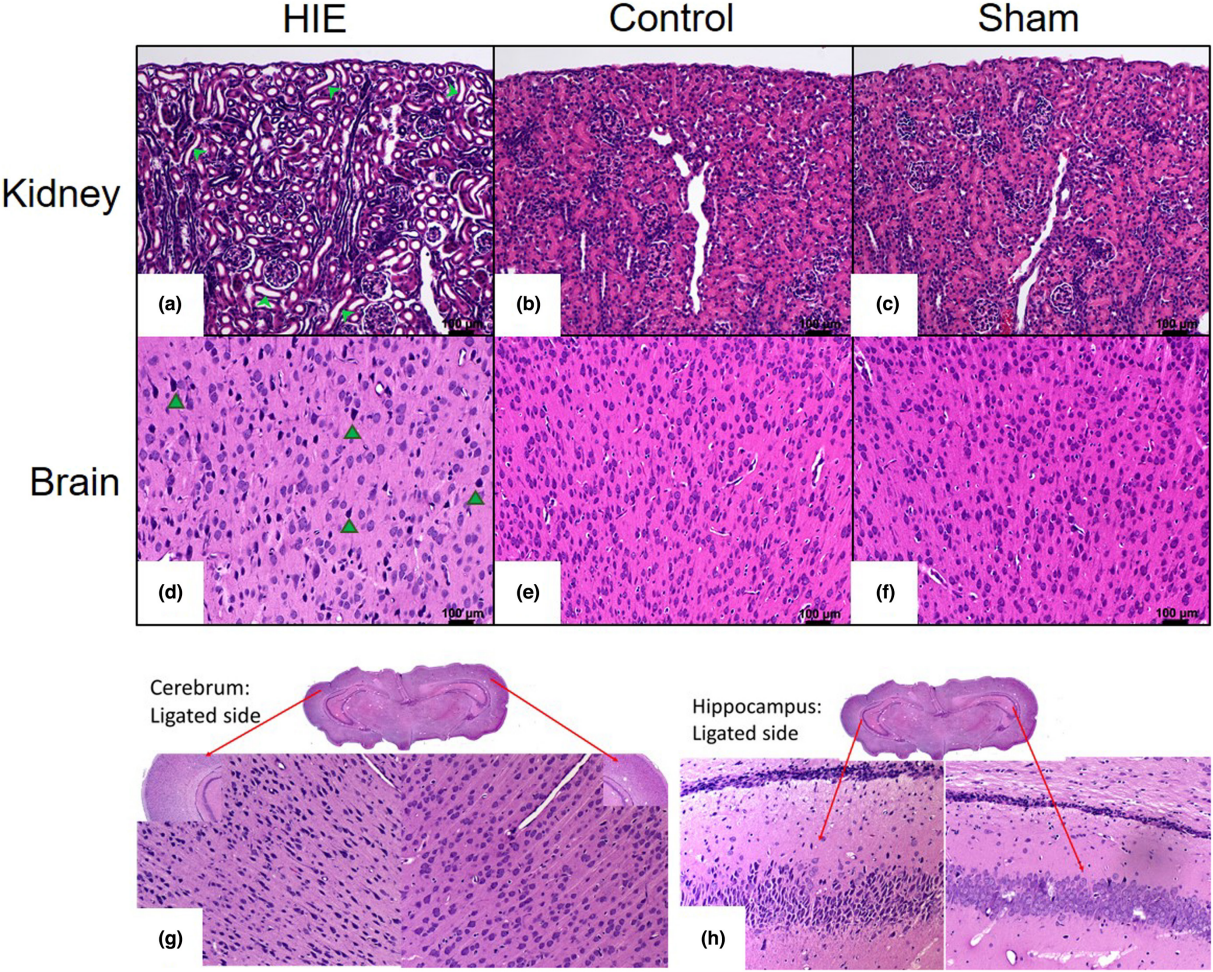
Photomicrographs of kidneys (a–c) and brains (d–f) from rat pups on day 7 following insult: (a) kidney sections from HIE insult showed damage to the proximal tubules of the kidney, which included tubular luminal dilatation (green arrow heads), simplified epithelial lining and brush border loss, without obvious loss of nuclei. No histological changes were observed in the control (b) or sham (c) groups. The cerebral cortex from pups receiving HIE insult (d) contained pyknotic neurons (green arrow heads) while the brains from the control (e) or sham (f) groups showed no histological changes (H&E stain, original magnification ×200). Following HIE procedure pyknotic neurons are visible in the cerebral cortex (g) and hippocampus (h) in the ligated hemisphere while the contralateral hemisphere did not ischemic change in the cerebral cortex or hippocampus (original magnification ×12.5; insets ×100 and ×200). *n* = 10–13 pups/treatment group.

Histopathological analysis of kidneys and brains were performed 7 days following hypoxic–ischemic injury to assess development of HIE and AKI (Table 1). Kidney injury was observed by light microscopy in 46% of the animals assessed. The changes were consistent with resolving acute tubular necrosis (ATN). The insult resulted in damage to the proximal tubules of the kidney as evidenced by tubular luminal dilatation (Figure 2a; green arrow heads) and loss of the brush border, as well as simplified epithelial lining, and increased mitotic activity. In general, the changes were mild, and in some cases focal, reflecting the fact that 7 days had elapsed from the time of the insult to harvesting of the organs for histological assessment. There was not obvious loss of nuclei and no casts were observed in the lumen, as commonly seen in early ATN. No histological changes were evident in the kidneys of the control (Figure 2b) or sham (Figure 2c) pups. Mitoses were present in the tubules of the kidneys of all groups as expected at this gestational age. The cerebral cortex of the pups exposed to the HIE insult contained pyknotic neurons (Figure 2d; green arrow heads) on the side corresponding to ligated carotid artery in 70% of the animals assessed, but there were no significant ischemic changes to neurons in the contralateral hemisphere (Figure 2g,h). No liquefactive necrosis, hemorrhage, obvious gliosis, or cyst formation was evident 7 days post‐injury, so no quantification of infarct volume was performed. The control (Figure 2e) and sham (Figure 2f) pups showed no significant histological changes in their brains on light microscopy. Of note, all of the subjects with histologically detectable kidney injury also showed pyknotic nuclei in the cerebral cortex.

**TABLE 1:** ‐ Histological evaluation of right kidney and cerebrum, evaluated on experimental day 7

Group	Sample	KIDNEY	TISSUE EDEMA	GRADE OF EDEMA	PROXIMAL/DISTAL TUBULES presence of dilated lumen with ragged brush border	TUBULES presence of mitoses in epithelial cells	PROXIMAL TUBULES presence of coarse vacuoles on surface of epithelium/lumen	DISTAL TUBULES fine vacuoles or degenerative changes to cytoplasm/luminal protrusions
		0 = normal 1 = abnormal	1 = cortex 2 = cortico‐medullary junction 3 = medulla 4 = 2 + 3 5 = all regions	1 = mild 2 = moderate 3 = severe	0 = none 1 = mild 2 = moderate 3 = severe	0 = none 1 = few 2 = moderate 3 = many	1 = few 2 = moderate 3 = many	1 = few 2 = moderate 3 = many
HIE	1	0	2	1	0	1	0	0
	2	1	2	1	1	1	0	0
	3	1	4	2	1	1	0	0
	4	0	2	1	0	1	0	0
	5	1	3	1	1	1	1	0
	6	0	4	1	0	1	0	0
	7	0	2	1	0	1	0	0
	8	0	2	1	0	1	0	0
	9	0	3	1	0	1	0	0
	10	0	4	1	0	1	0	0
	11	1	3	1	2	1	1	1
	12	1	4	1	2	2	0	0
	13	1	2	1	2	1	0	0
Nothing	1	0	4	1	0	1	0	0
	2	0	2	1	0	1	0	0
	3	0	2	1	0	1	0	0
	4	0	2	1	0	1	0	0
	5	0	2	1	0	1	0	0
	6	0	4	1	0	1	0	0
	7	0	2	1	0	1	0	0
	8	0	2	1	0	1	0	0
	9	0	2	1	0	1	0	0
	10	0	2	1	0	1	0	0
	11	0	3	1	0	1	0	0
Sham	1	0	3	1	0	1	0	0
	2	0	2	1	0	1	0	0
	3	0	4	1	0	1	0	0
	4	0	0		0	1	0	0
	5	0	2	1	0	1	0	0
	6	0	4	2	0	1	0	0
	7	0	2	1	0	1	0	0
	8	0	2	1	0	1	0	0
	9	0	2	1	0	1	0	0
	10	0	2	1	0	1	0	0

Surgery	Sample	BRAIN cerebrum	Foci cortical vacuoles	Hypoxic neurons	Hypoxic neurons	Drop out of neurons	Cortical infarcts or cystic change
		0 = normal 1 = abnormal	0 = absent 1 = present	0 = no 1 = yes	1 = few 2 = moderate 3 = many	1 = few 2 = moderate 3 = many	0 = absent 1 = present
HIE	1	1	1	1	1	1	0
	2	1	1	1	1	1	0
	3	1	1	1	1	1	0
	4	1	0	1	1	0	0
	5	1	0	1	1	0	0
	6	0	0	0	0	0	0
	7	0	0	0	0	0	0
	8	1	0	1	1	0	0
	9	0	0	0	0	0	0
	10	1	1	1	1	0	1
	11	1	1	1	1	0	0
	12	1	0	1	1	0	0
	13	1	0	1	2	0	0
Nothing	1	0	1	0	0	0	0
	2	0	1	0	0	0	0
	3	0	1	0	0	0	0
	4	0	1	0	0	0	0
	5	0	0	1 (scattered)	1	0	0
	6	0	0	1 (scattered)	1	0	0
	7	0	0	1 (scattered)	1	0	0
	8	0	0	0	0	0	0
	9	0	0	1 (scattered)	1	0	0
	10	0	1	1 (scattered)	1	0	0
	11	0	1	1 (scattered)	1	0	0
Sham	1	0	1	0			
	2	0	1	0			
	3	0	1	0			
	4	0	1	0			
	5	0	0	1 (scattered)	1	0	0
	6	0	0	1 (scattered)	1	0	0
	7	0	0	1 (scattered)	1	0	
	8	0	0	1 (scattered)	1	0	0
	9	0	0	1 (scattered)	1	0	0
	10	0	0	1 (scattered)	1	0	0

*Note*: Additional kidney parameters assessed and determined to be absent in all samples: PROXIMAL TUBULES ‐ Surface blebs or drop out of epithelium; PROXIMAL TUBULES ‐ presence of coarse vacuoles into cytoplasm of epithelial cells in region of corticomedullary junction; TISSUE INFLAMMATION; GLOMERULI – fibrosis. Colored cells highlight specimens designated as abnormal upon histopathological evaluation.

**FIGURE 3:**
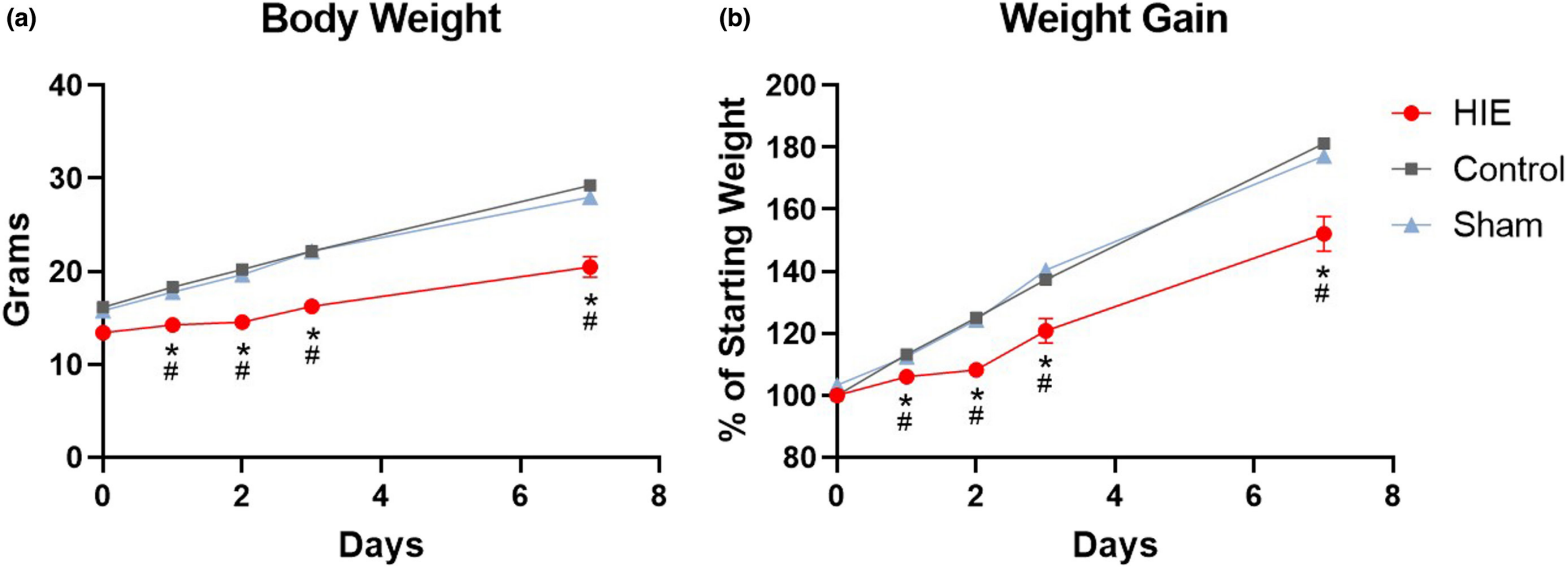
Body weight (a) and percent weight gain above D0 body weight (b) of pups receiving either HIE injury, sham surgery, or no intervention control, at 0, 1, 2, 3, and 7 days from start of experiment. Symbols represent SEM of *n* = 6 pups/treatment group/time point. *significantly different (*p* < 0.05) from time point matched controls. ^#^Significantly different (*p* < 0.05) from time point matched shams.

As a measure of health condition, morbidity, and an indicator of toxicity, body weight was recorded starting at 7 days of age, the time of HIE insult, sham surgery, or control handling (experimental day 0), and continued daily for the duration of the experiment (Figure 3, Table 2). No significant differences were observed in starting weight between sexes. Male and female rat pups weighed on average 14.9 g and 15.3 g, respectively, and average weights of pups in HIE, sham surgery, and control groups were 13.4 g, 15.8 g, and 16.5 g, respectively, differences that were not statistically significant (Figure 3a, Table 2). Both absolute weight and weight gain were significantly reduced in HIE pups compared to control and sham pups, starting at day 1 and continuing throughout the course of the experiment until sacrifice 7 days later. Seven days post‐start of the experiment, controls were 81.2% above their starting weight compared to 52.1% in HIE pups (Figure 3b, Table 2). The effect of HIE on weight gain did not differ between sexes in this study with gains at D7 averaging 51.3% and 53.0% for male and female pups, respectively. No significant reduction in weight gain was observed in pups receiving sham surgery.

**TABLE 2:** *p* values for average pup weight gain (absolute and relative to starting weight) for comparisons between experimental groups

*p* values: Grams of weight gained		
Days	HIE versus Control	HIE versus Sham
0	0.0001	0.0036
1	0.0003	<0.0001
2	0.0003	0.0001
3	0.0004	0.0004
7	0.0003	0.0004

*p* values: % Gain		
Days	HIE versus Control	HIE versus Sham
1	0.0285	0.0201
2	0.0023	0.0001
3	0.0049	0.0016
7	0.0033	0.0005

**FIGURE 4:**
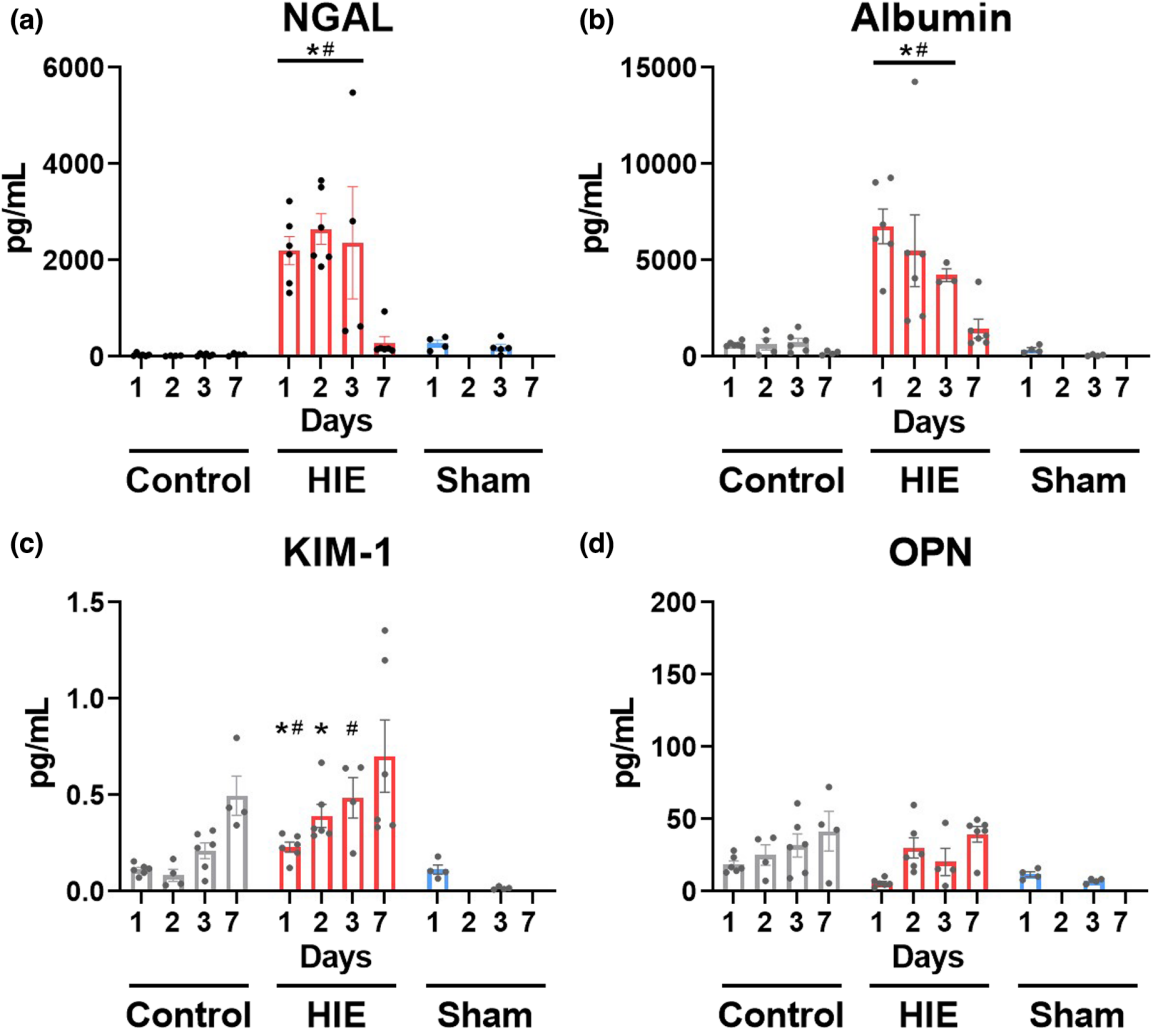
Urinary biomarkers of AKI, collected from pups receiving either HIE injury, sham surgery, or no intervention control, at 1, 2, 3, and 7 days from start of experiment. Protein abundance in urine was determined via ELISA. Bars represent SEM of *n* = 4–6 pups/treatment group/time point. Data points represent individual animals. *Significantly different (*p* < 0.05) from time point matched controls. ^#^Significantly different (*p* < 0.05) from time point matched shams.

Biomarkers of AKI were measured in urine collected from pups, starting immediately prior to the start of the experiment, and at days 1, 2, 3, and 7 (Figure 4, Table 3). Day 0 results are presented in Table S1 and did not significantly differ from control values. Pups receiving HIE had significantly elevated urinary levels of NGAL and albumin compared to sham or controls days 1–3 post‐insult, returning to control levels day 7 post‐insult (Figure 4a,b, Table 3). KIM‐1 was significantly increased compared to control and sham groups for 2 days post‐HIE. By day 3 elevations in KIM‐1 in HIE pups were statistically different from sham but not control levels (Figure 4c, Table 3). HIE insult did not significantly elevate osteopontin levels above control and sham groups at any time measured in the experiment (Figure 4d, Table 3). Hypoxia exposure alone without carotid artery ligation resulted in a non‐statistically significant elevation in NGAL on day 1, which occurred to a lesser extent than HIE injury, and did not impact the other biomarkers examined (Figure S1).

**TABLE 3:** *p* values for average biomarker abundance in urine collected on days 1–7 following intervention

	Days	HIE versus control (*p* values)	HIE versus sham (*p* values)
NGAL	1	**0.0002**	**0.036**
	2	**<0.0001**	
	3	**0.0004**	**0.023**
	7	0.98	
Albumin	1	**<0.0001**	**<0.0001**
	2	**0.003**	
	3	**0.0188**	**0.003**
	7	0.077	
KIM‐1	1	**0.002**	**0.023**
	2	**0.013**	
	3	0.19	**<0.0001**
	7	0.45	
Osteopontin	1	0.5266	0.5486
	2	0.9816	
	3	0.7174	0.1137
	7	0.9992	

The bold values indicate statistical significance (*p* < 0.05).

**FIGURE 5:**
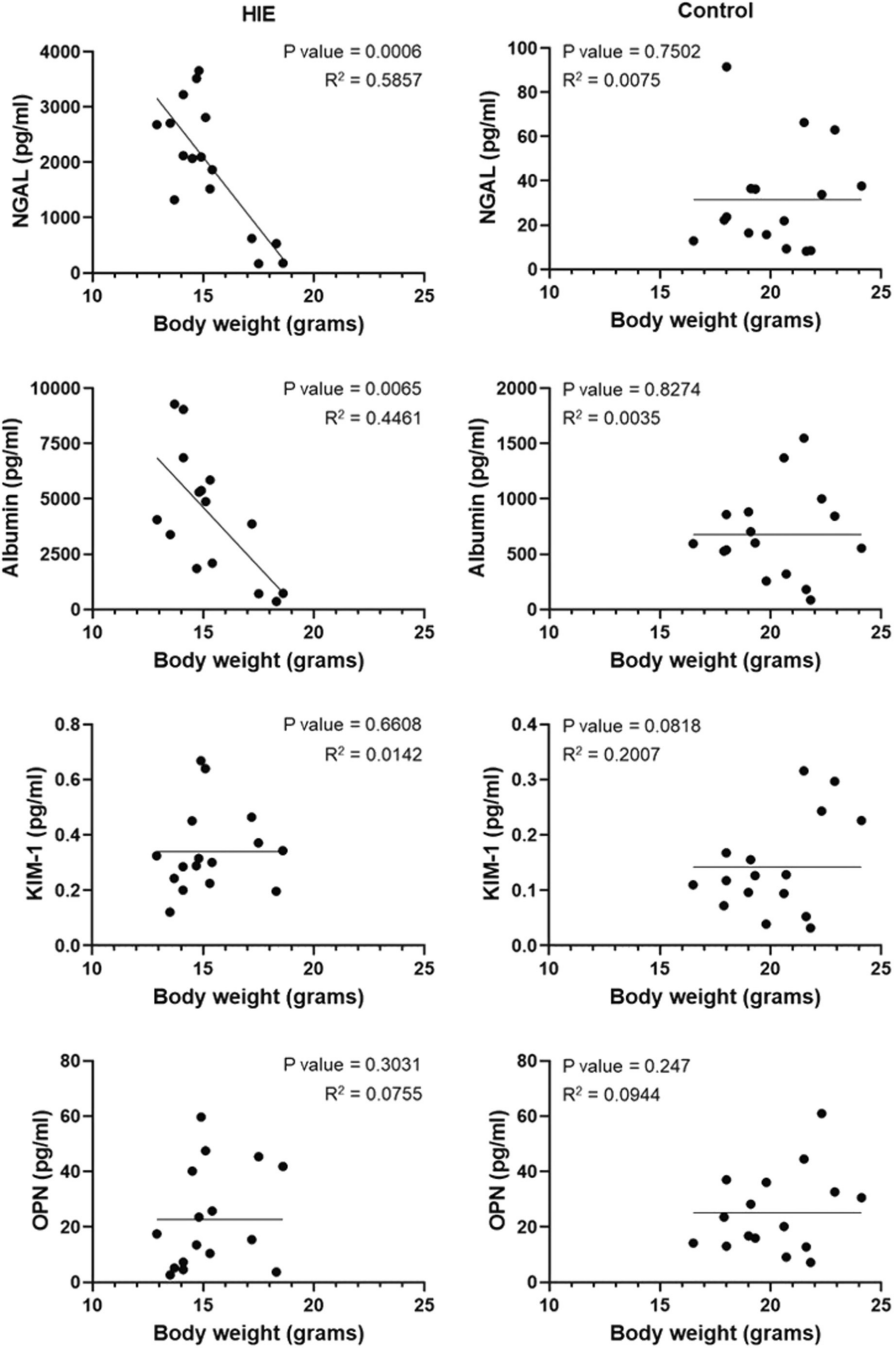
Correlation between urinary biomarkers of AKI and body weight within individual pups receiving either HIE injury or no intervention control, at 1, 2, and 3, days from start of experiment. Data points represent a single time point for an individual animal. Lines represent linear fits of data sets associated with *p* and R^2^ values. *n* = 4–6 pups/treatment group/time point.

In order to determine whether urinary biomarkers of AKI correlated with health status for an individual, each analyte was correlated with the corresponding body weight recorded at the time of urine collection for each subject. Days 1–3 were included in the analysis, as significant changes in analyte abundance occurred during this time frame. Body weight was significantly inversely correlated with the abundance of NGAL and albumin in animals receiving HIE injury but not in non‐injured control animals (Figure 5). No significant correlations were observed with KIM‐1 or OPN in both HIE and non‐injured control animals.

## 4 DISCUSSION

The Rice–Vannucci model is a widely utilized experimental approach for studying perinatal HIE and has been used extensively for identifying mechanisms of brain injury related to birth asphyxia (Millar et al., 2017; Rice et al., 1981; Vannucci & Vannucci, 1997, 2005). Less well studied, but of equal importance, are the renal pathologies associated with this model, since it potentially replicates a component of the kidney injury that is observed in up to 40% of neonates that have experienced perinatal asphyxia. Additionally, the development of AKI strongly contributes to poorer short‐term outcomes as well as negative long‐term consequences in those affected (Harer et al., 2017; Tanigasalam et al., 2016). Despite this clinical evidence, few animal models of these short‐ and long‐term outcomes currently exist. Xu et al. has recently published a histological characterization of the pathological processes occurring in the kidney resulting from the HIE model, reporting significant swelling of tubular epithelial cells, interstitial edema, and necrotic changes in the renal cortex, as well as disruption of glomerular filtration barriers (Xu et al., 2017). Their histological characterization showed this occurred between 3 and 72 h following the Rice–Vannucci HIE procedure. Our findings showing that AKI changes had largely resolved by day 7 are not unexpected given the brush border loss/ regeneration cycle that occurs after ischemic injury (Venkatachalam et al., 1978). Moreover, the absence of severe ischemia in the brain is also not unexpected given that even after bilateral carotid artery ligation in rats, other investigators have found absence of severe ischemia due to the presence of anastomotic channel function to effectively perfuse the forebrain (Brown, 1966). Of note, although we were able to detect short‐term kidney injury with this model, an important next step will be to determine whether kidney dysfunction persists into adulthood in these animals.

Here we further expand the utility of this model by presenting a noninvasive approach to longitudinally track the progression of AKI through urinary biomarkers, which can be used to monitor the efficacy of therapeutic interventions. Our histological outcomes displayed evident kidney pathology that were preceded by elevation of established urinary biomarkers of AKI.

The clinically established method for diagnosing AKI is via an elevation of serum creatinine, which is cleared through the glomerulus, and is therefore a proxy of glomerular filtration rate and renal functioning (Xu et al., 2018). It has served as an important indicator of injury or toxicity to the kidney that would disrupt or impair this process. However, injury to the kidney can occur before a decrease in filtration rate becomes detectable; therefore, the need exists to develop biomarkers for clinical use, not only in adults, but also in children and neonates, that are more sensitive in detecting renal injury and which are rapidly responsive (Edelstein, 2017; Sandokji & Greenberg, 2020). This is critical because early detection provides additional time and opportunity for intervention and enables treatment to occur at an earlier stage of pathogenesis (Edelstein, 2017). Several biomarkers have been examined in both humans and in preclinical animal studies and meet the criteria of being sensitive, specific, and predictive of AKI (Bolisetty & Agarwal, 2011; Edelstein, 2017; Mishra et al., 2003; Sandokji & Greenberg, 2020; Vaidya et al., 2006). These include the analytes presented in this manuscript.

We present an experimental approach that utilizes biomarkers to enable early and sensitive detection of AKI resulting from HIE. Urinary NGAL has been established as an early responding biomarker of AKI, not only in rats following early ischemic AKI and in mice from cisplatin toxicity (Mishra et al., 2003, 2004), but also in both adult and pediatric patients after cardiac surgery (Mishra et al., 2005; Wagener et al., 2006), and in critically ill children with heterogeneous illness (Zeid et al., 2019). In both preclinical experiments and in clinical studies of pediatric cohorts, NGAL elevation preceded rises in other early responding markers (Mishra et al., 2003), and this is in line with our observations showing significant increases in NGAL as early as 1 day following injury, the earliest time point assayed in this study. Urinary albumin, also becomes present in the urine in response to stress to renal tubules and after various glomerulopathies and in mice has been detected as early as 4 h after AKI induction (Ware et al., 2011). Accordingly, we also observed early increases in urinary albumin, detected at 1 day following HIE. In investigations of another biomarker of renal injury, KIM‐1, studies in pediatric patients found that this marker was elevated after cardiac surgery; however, this was delayed compared to NGAL detection (Devarajan, 2011; Dong et al., 2017). In line with this, the time dependent increase in KIM‐1 was affected by AKI, becoming elevated within the first 3 days and reaching its greatest values by 7 days following HIE induction, a time point in which NGAL and albumin levels returned to baseline. This supports the use of a panel of biomarkers that includes not only those which can be used to rapidly identify the initial development of AKI but also those that are sensitive to lasting impairments. Interestingly osteopontin did not respond to AKI in our HIE model, and recovery from sham surgery may have impacted biomarker levels, specifically at the 3 day time point. Determination of the specific mechanisms that relate the responsiveness of these biomarkers to the nature and extent of renal damage in this model is beyond the scope of this study, but provides potential opportunities for further investigation. These biomarkers differ from other measures of functional output of the kidney, such as serum creatinine, in that they are responsive to and, ideally, should be able to differentiate between, a variety of factors including tubular injury, glomerulonephritis, and interstitial nephritis (Edelstein, 2017). The differential responses of the biomarkers in our study would enable these types of mechanistic investigations.

Our findings of a correlative relationship between biomarker changes and weight gain (or lack thereof) also provide potential for their use in AKI prediction, providing the possibility of relating the extent of their elevation to the severity of AKI. This relationship between overall body condition and renal injury mirrors the increased morbidity and poorer outcomes observed in neonates that develop AKI as an aspect of HIE/perinatal asphyxia (Cavallin et al., 2020; Robertsson Grossmann et al., 2022). Importantly, biomarker sensitivity to injury severity could potentially serve as an indicator of efficacy for novel therapeutics in preventing or reversing AKI. The relationship between HIE and AKI is well documented in the clinic (Durkan & Alexander, 2011); however, mechanistic studies investigating the processes that lead to this outcome, such as oxidative stress, are less well understood and could be aided by the utilization of urinary biomarkers in this rat model. One of the limitations of this study is that although we did not observe any differences in outcomes between sexes, larger group sizes are needed to assess the role of sex on sensitivity to AKI. Further investigations should consider sex as a biological variable, include more detailed comparisons of urinary biomarker change with kidney functioning by serum creatine, and assess this model for any lasting long‐term effects or susceptibility to kidney disease later in life.

## 5 CONCLUSIONS

The Rice–Vannucci neonatal model of HIE produces an AKI. This model provides a feasible experimental design to examine the renal injury resulting from HIE. Importantly, the utilization of a panel of biomarkers that are rapid and sensitive in detecting and monitoring AKI provides a valuable system to investigate the effectiveness of potential therapeutic interventions and their influence on morbidity and mortality.

## AUTHOR CONTRIBUTIONS

All authors contributed to the study conception and design. Material preparation, data collection, and analysis were performed by Angela M Groves, Carl J Johnston, and Alison L Kent. The first draft of the manuscript was written by Angela M Groves and all authors contributed revisions critically important for its intellectual content. All authors read and approved the final manuscript.

## FUNDING INFORMATION

Partial financial support was received from NIH R01 HL144776 to GP, NIH R01 HL091968 to MOR, and P30ES001247.

## CONFLICT OF INTEREST

The authors have no relevant financial or non‐financial conflicts of interest to disclose.

## Supporting information


Table S1
Figure S1
