# Supplementary material for: Neonatal hypoxic ischemic encephalopathy increases acute kidney injury urinary biomarkers in a rat model
Source: Physiol Rep. 2022 Dec 21;10(24):e15533. doi: 10.14814/phy2.15533 (PMC9768655; doi:10.14814/phy2.15533)

**Supplementary Table 1** Day 0 values for urinary biomarkers in pups receiving HIE intervention. Expressed as average values in pg/ml, n = 4-6 pups.

| **D0 values: HIE subjects** | | |
| --- | --- | --- |
|  | **Average** | **SEM** |
| **NGAL** | 120.64 | 38.67 |
| **Albumin** | 1967.29 | 291.96 |
| **KIM-1** | 0.12 | 0.03 |
| **OPN** | 4.50 | 1.29 |

**Supplementary Figure 1** Urinary biomarkers of AKI, collected from pups receiving hypoxia alone (8% oxygen for 120 minutes) at 1day from the start of the experiment. Protein abundance in urine was determined via ELISA. Bars represent SEM of n=4 pups/treatment group. Data points represent individual animals. NS=Not Significantly different (P>0.05) from time point matched controls.


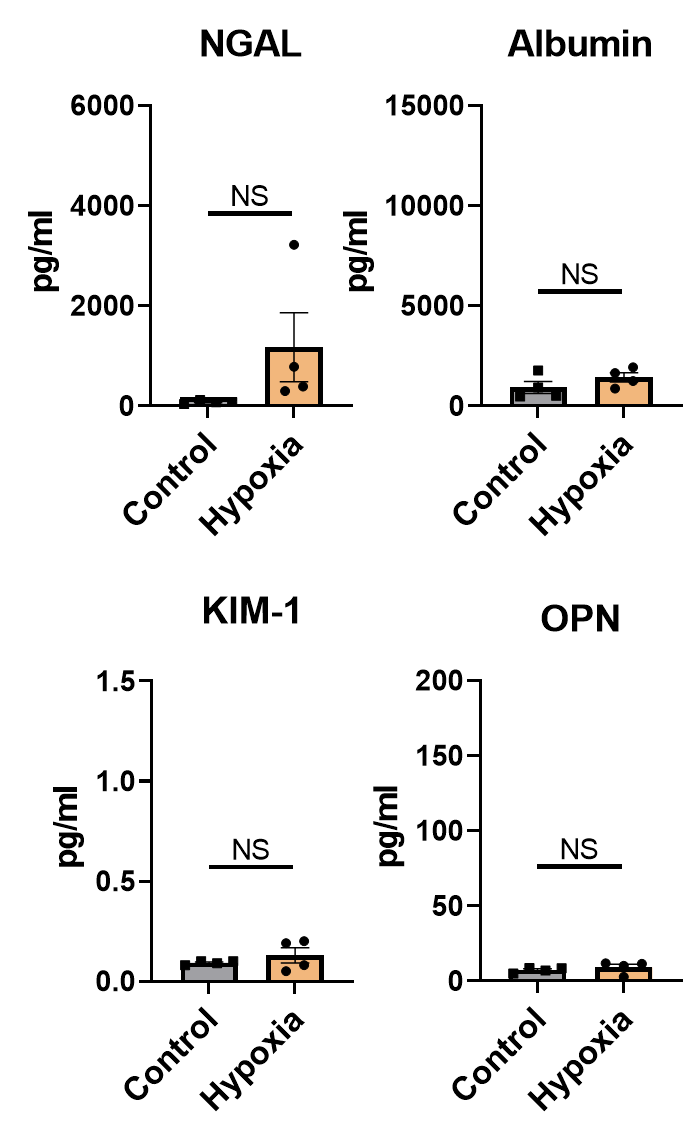

Supplement: Supplementary file 1 — Table S1 Figure S1 [file PHY2-10-e15533-s001.docx]
